# NRT1.1s in plants: functions beyond nitrate transport

**DOI:** 10.1093/jxb/erz554

**Published:** 2019-12-13

**Authors:** Wei Wang, Bin Hu, Aifu Li, Chengcai Chu

**Affiliations:** 1 State Key Laboratory of Plant Genomics, Institute of Genetics and Developmental Biology, the Innovative Academy of Seed Design, Chinese Academy of Sciences, Beijing, China; 2 Guangdong Laboratory of Lingnan Modern Agriculture, Guangzhou, China; 3 Nanjing Agricultural University, China

**Keywords:** Arabidopsis, nitrate transporter, nitrogen use efficiency, NRT1.1, rice, signal transduction

## Abstract

Arabidopsis AtNRT1.1 (CHL1/AtNPF6.3) is the first nitrate transporter identified in plants and was initially found to play a role in nitrate uptake and transport. AtNRT1.1 also displays auxin transport activity and mediates nitrate-modulated root development, suggesting that it has transport capacity for multiple substrates. Subsequent work revealed that AtNRT1.1 can respond to environmental nitrate fluctuations by altering its nitrate transport activity, modulated by phosphorylation, leading to the critical finding that AtNRT1.1 acts as a transceptor for nitrate sensing. Recent studies have revealed how OsNRT1.1B, the functional homologue of AtNRT1.1 in rice, mediates nitrate signal transduction from the plasma membrane to the nucleus, and how OsNRT1.1B integrates the nitrate and phosphate signaling networks. OsNRT1.1B has also been shown to be involved in regulating the root microbiota to facilitate organic nitrogen mineralization in soil, thus mediating plant–microbe interactions. Furthermore, the divergent functions of OsNRT1.1A and OsNRT1.1B in regulating nitrogen use in rice suggest that the function of NRT1.1 is still far from fully understood. In this review, we focus on the most recent progress on the molecular mechanisms of NRT1.1s in plants, with the aim of providing an up-to-date view of the versatile functions of NRT1.1 in nitrogen utilization in plants.

## Teaser

This review summarizes the recent progress of research on the functions of NRT1.1 members in plants, with the aim of providing an up-to-date view of the role of NRT1.1s in nitrogen utilization by plants.

## Introduction

Nitrate is one of the most important nitrogen sources for plants due to its function as both a nutritional component and a signaling molecule (Wang *et al.*, 2018*a*). Investigating the molecular mechanisms of nitrate utilization and signaling is critical for studies of mineral nutrition in plants. It would also provide a vital theoretical basis for improving nitrogen use efficiency (NUE) in crops, thus contributing to sustainable agriculture. The identification of the first nitrate transporter, AtNRT1.1 (CHL1/AtNPF6.3), in Arabidopsis represents an important milestone on the road to understanding the molecular basis of nitrate use in plants (Tsay *et al.*, 1993). By performing large-scale screening of an Arabidopsis T-DNA mutant library using chlorate, a toxic analog of nitrate, Tsay *et al.* (1993) successfully identified AtNRT1.1. AtNRT1.1 belongs to the NITRATE TRANSPORTER 1/PEPTIDE TRANSPORTER FAMILY (NPF), which exists across all organisms and was initially characterized as a transporter for peptides. Initially, it was found that AtNRT1.1 is involved in mediating nitrate uptake by the roots as well as root-to-shoot nitrate transport in Arabidopsis. The location of AtNRT1.1 in the epidermal and vascular cells also strongly supported its function in nitrate uptake and translocation (Huang *et al.*, 1996; Leran *et al*., 2013). The homologs of AtNRT1.1 in rice and maize also display nitrate transport activity, indicating a conserved function of NRT1.1 in nitrate uptake and/or transport across different species (Hu *et al.*, 2015; Wen *et al.*, 2017). However, a series of research studies on NRT1.1 have provided new understanding of its multiple functions in both Arabidopsis and rice, demonstrating that the function of NRT1.1 is more than that of a simple nitrate transporter. In this review, we summarize the various functions of NRT1.1s, including auxin transport activity to modulate root development, nitrate signal transduction, and the integration of nutrient signals, as well as a regulatory role in plant–microbe interaction, with the aim of providing an comprehensive and up-to-date overview of the functions of NRT1.1 in plants.

## AtNRT1.1 possesses auxin transport activity to modulate lateral root development

Auxin has long been considered to be a dominant regulator for lateral root formation (Himanen *et al.*, 2002; Fukaki and Tasaka, 2009). Besides nitrate transport activity, AtNRT1.1 also displays auxin transport activity and regulates lateral root growth by modulating auxin transport activity in a nitrate-dependent manner in Arabidopsis (Krouk *et al.*, 2010). When little or no nitrate is available, AtNRT1.1 can exhibit auxin transport activity and prevent the accumulation of auxin in the lateral root tip, which inhibits the elongation of the lateral root. Conversely, the auxin transport activity of AtNRT1.1 is inhibited in the presence of nitrate, resulting in the accumulation of auxin in the lateral root tip and the elongation of the lateral root (Krouk *et al.*, 2010). Further work demonstrated that phosphorylation of threonine residue 101 (Thr101) in AtNRT1.1 is necessary for auxin transport activity and for the inhibition of lateral root elongation in conditions of low nitrate availability (Bouguyon *et al.*, 2015). These results indicate a close relationship between nutrient and phytohormone signaling during plant development. However, there is a question about how the function of AtNRT1.1 is compatible with the down-regulation of *AtNRT1.1* under conditions of little or no nitrate at the whole-root level and in the lateral root primordium. Recent studies have demonstrated that nitrate can regulate *AtNRT1.1* at the post-transcriptional level in a tissue-specific manner. Nitrate can increase the transcription and mRNA accumulation of *AtNRT1.1* in the lateral root primordium, but represses the accumulation of AtNRT1.1 protein in the lateral root primordium, with a concomitant increase of auxin accumulation to promote lateral root elongation (Bouguyon *et al.*, 2016). These findings further suggest a critical role of AtNRT1.1 in regulating the root system architecture. Thus, it is possible that NRT1.1 is able to transport multiple substrates, indicating a critical role of NRT1.1 in the integration of environmental and physiological information in response to nutrient availability.

## AtNRT1.1 has dual-affinity transport activity for nitrate

**Fig. 1.**
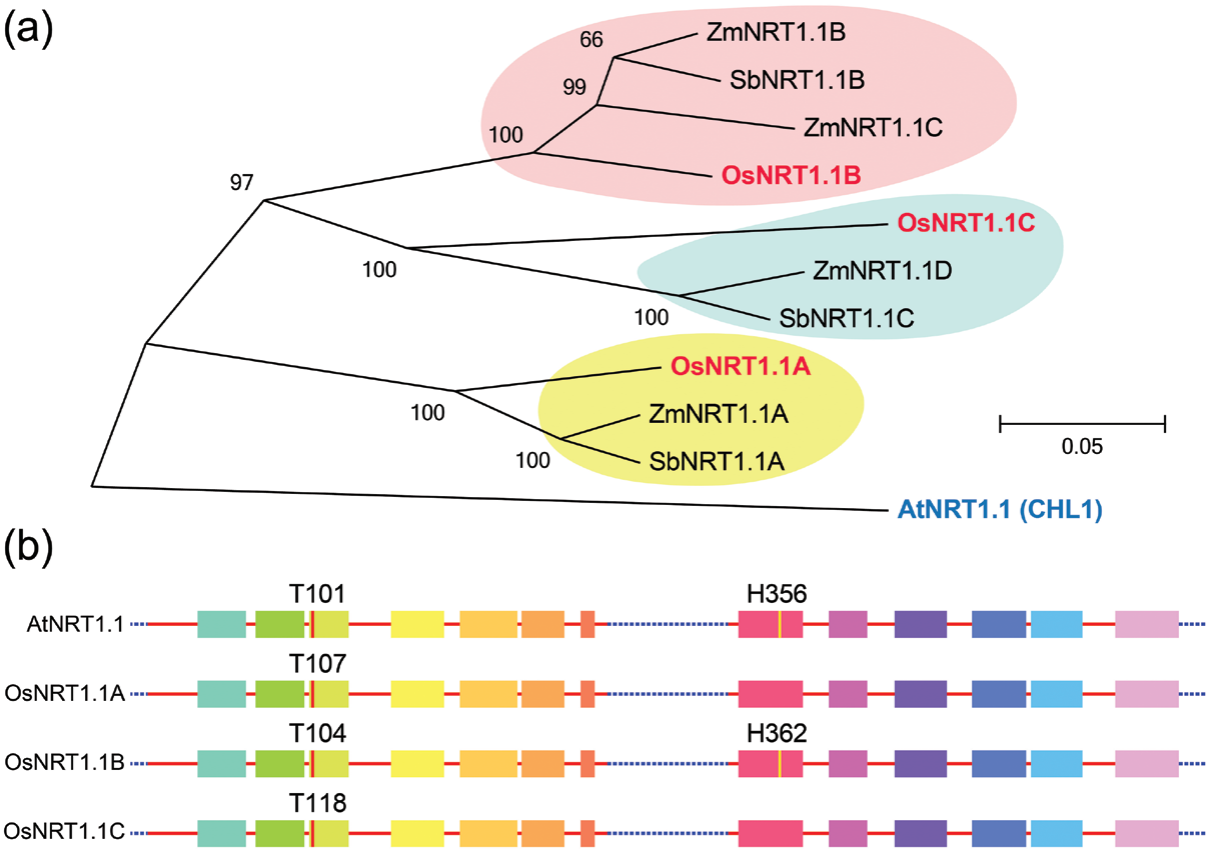
Protein sequence analysis of NRT1.1 members in the eudicotyledonous plant Arabidopsis and three monocotyledonous grass species. (A) Phylogeny of NRT1.1 members in Arabidopsis, rice, maize, and sorghum. The phylogenetic tree was constructed by MEGA6 software using the neighbor-joining method with 1000 bootstrap replicates (Tamura *et al.*, 2013). The accession numbers of the NRT1.1 proteins for the different plant species are as follows: AtNRT1.1 (AT1G12110), OsNRT1.1A (LOC_Os08g05910), OsNRT1.1B (LOC_Os10g40600), OsNRT1.1C (LOC_Os03g01290), ZmNRT1.1A (GRMZM2G086496_P01), ZmNRT1.1B (GRMZM2G161459_P02), ZmNRT1.1C (GRMZM2G112154_P01), ZmNRT1.1D (GRMZM2G161483_P01), SbNRT1.1A (Sb07g003690), SbNRT1.1B (Sb01g029470) and SbNRT1.1C (Sb01g050410). At, *Arabidopsis thaliana*; Os, *Oryza sativa*; Zm, *Zea mays*; Sb, *Sorghum bicolor*. (B) Alignment of conserved amino acid residues in NRT1.1 members in Arabidopsis and rice. T101 is conserved in NRT1.1 proteins in both Arabidopsis and rice, while the nitrate-binding site H356 is absent in OsNRT1.1A and OsNRT1.1C.

To cope with fluctuations in external nitrate concentrations, plants have evolved different transport systems to adjust their nitrate uptake capacity (Miller *et al.*, 2007). There are two uptake systems within plants: the high-affinity system works when the nitrate concentration in the soil is low (<1 mM), whereas the low-affinity system operates in conditions of high nitrate (>1 mM) (Dechorgnat *et al.*, 2011). NRT1 members are thought to normally function as components of the low-affinity uptake system under high nitrate concentrations; however, AtNRT1.1 exhibits dual-affinity nitrate transport activity in both low- and high-nitrate concentrations (Liu *et al.*, 1999). Interestingly, subsequent research showed that the switch of AtNRT1.1 between the high- and low-affinity nitrate transport systems is modulated by phosphorylation of Thr101 in response to fluctuations in the nitrate level. When Thr101 is phosphorylated by CBL-INTERACTING PROTEIN KINASE 23 (CIPK23), it functions as a high-affinity transporter in response to low nitrate availability; under high nitrate conditions, it is dephosphorylated and works as a low-affinity nitrate transporter. Studies of the crystal structure of AtNRT1.1 suggested that phosphorylation of Thr101 may change the affinity of AtNRT1.1 for nitrate via decoupling the dimer of AtNRT1.1 (Parker and Newstead, 2014; Sun *et al.*, 2014). When nitrate is sufficient, AtNRT1.1 is dephosphorylated at Thr101, forms a dimer with decreased structural flexibility, and mediates low-affinity uptake of nitrate. While external nitrate is not abundant, AtNRT1.1 is phosphorylated, which decouples the dimer, and it functions as a high-affinity transporter with high structural flexibility to acquire a small account of nitrate from the soil. Furthermore, studies of the crystal structure also revealed that His356 of AtNRT1.1 is critical for nitrate recognition. Mutation of His356 to Ala356 abolished both the high- and low-affinity nitrate transporter activity of AtNRT1.1, suggesting that His356 is required for nitrate binding (Parker and Newstead, 2014; Sun *et al.*, 2014). Remarkably, His356 is not well conserved among NRT1 members, suggesting that other NRT1 members recognize and transport nitrate via an unknown mechanism (Fig. 1). However, evaluation of the rate of nitrate influx of *atnrt1.1* mutant plants shows that the high-affinity nitrate influx mediated by AtNRT1.1 is not significant *in planta* (Glass and Kotur, 2013; Wen and Kaiser, 2018). This inconsistency might be due to differences in measuring time, membrane chemistry, and surface:volume ratios between the heterologous *Xenopus* oocyte system and plants (Krapp *et al.*, 2014; Wei *et al.*, 2018; Chang *et al.*, 2019). Interestingly, AtNRT1.1 is known to be necessary for plant growth under conditions of low nitrate supply (Ye *et al.*, 2019), suggesting that AtNRT1.1 may act as a nitrate sensor rather than a nitrate transporter under low nitrate conditions.

## AtNRT1.1 acts as a nitrate transceptor

Besides being a fundamental inorganic nitrogen source for most plants, nitrate serves as a signal molecule that triggers downstream nitrogen responses in regulating gene expression, metabolism, and developmental processes of plants. Remarkable progress was achieved by work showing that AtNRT1.1 also acts as a nitrate transceptor to perceive different levels of external nitrate in Arabidopsis (Ho *et al.*, 2009). As mentioned above, AtNRT1.1 is phosphorylated by CIPK23 under low nitrate conditions, which triggers a low-level nitrate response (i.e. high-affinity uptake). When external nitrate is sufficient, AtNRT1.1 is dephosphorylated, leading to a high-level nitrate response. Moreover, AtNRT1.1 has been shown to control not only the nitrate response but also the feedback regulation of gene expression in response to long-term high nitrate supply (Munos *et al*., 2004; Bouguyon *et al.*, 2015) and the root foraging process in response to nitrate (Remans *et al.*, 2006; Krouk *et al.*, 2010). These different roles of AtNRT1.1 may depend on the fact that AtNRT1.1 can activate multiple independent nitrate sensing/signaling events. However, the detailed mechanisms of the different AtNRT1.1-mediated different signaling pathways are largely unknown.

In addition to the nitrate transceptor AtNRT1.1, AtNLP7 is a master regulator involved in the activation of nitrate-responsive genes in Arabidopsis. The cytoplasmic–nuclear shuttling of AtNLP7, which is stimulated by nitrate, transmits the nitrate signal from the cytoplasm to the nucleus (Marchive *et al.*, 2013). A more recent study revealed that calcium-dependent protein kinases (CPK10/30/32) phosphorylate AtNLP7 at Ser205, depending on nitrate-induced calcium influx (Liu *et al.*, 2017). Dephosphorylation of AtNLP7 is retained in the cytosol, indicating that calcium may function as a secondary messenger to regulate CPK activities and activate nitrate signaling. Although a nitrate signaling pathway including nitrate sensing and a downstream nitrate response has emerged, the essential factors connecting the NRT1.1 transceptor and the master regulator NLP have yet to be identified.

## The OsNRT1.1B–OsSPX4 module transduces the nitrate signal from the plasma membrane to the nucleus

**Fig. 2.**
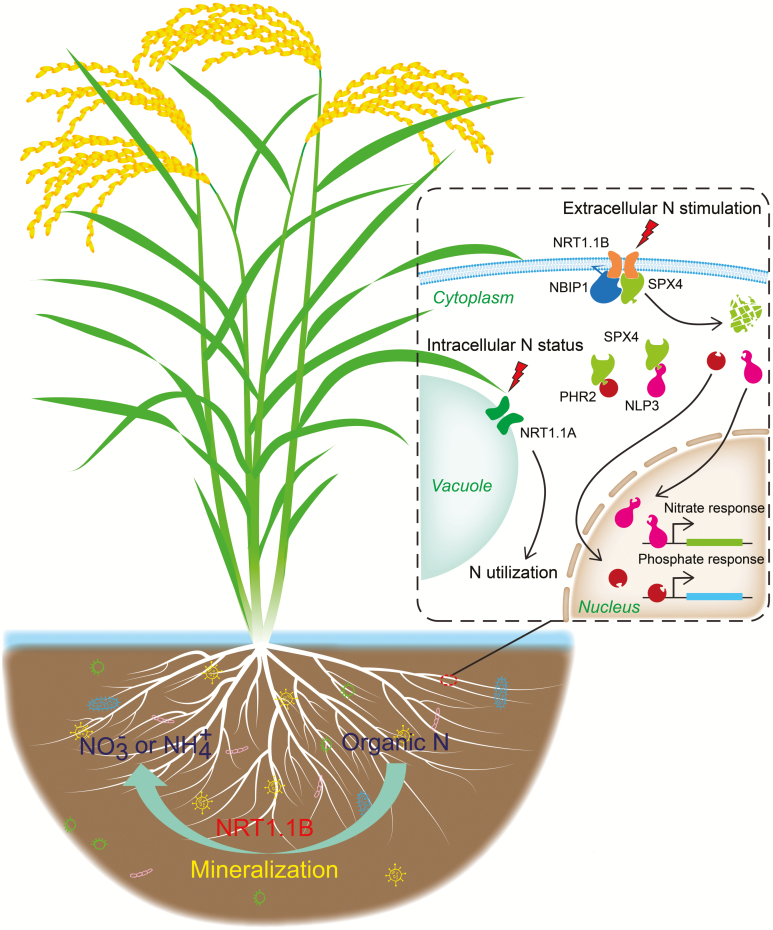
Integrative model illustrating the roles of NRT1.1s in rice. Plasma membrane-localized OsNRT1.1B (NRT1.1B) functions as a nitrate sensor in response to external nitrate, while tonoplast-localized OsNRT1.1A (NRT1.1A) possibly acts as an intracellular nitrate sensor in the perception of the cell’s nitrogen status. The NRT1.1B–OsSPX4 (SPX4) module is a key node involved in integrating nitrate and phosphate signals in rice. In the presence of nitrate, NRT1.1B senses external nitrate and recruits an E3 ligase, NBIP1, to promote SPX4 ubiquitination and degradation via the 26S proteasome, thereby releasing OsPHR2 and OsNLP3 (NLP3) to concurrently activate downstream phosphate and nitrate responses, respectively. In addition, NRT1.1B can change the rhizosphere microenvironment by modulating root microbes related to nitrogen transformation.

In rice, OsNRT1.1B/OsNPF6.5, the functional homologue of AtNRT1.1, has also been demonstrated to sense an external nitrate signal (Hu *et al.*, 2015). Interestingly, our recent study revealed that OsNRT1.1B can physically interact with OsSPX4, the critical repressor protein involved in phosphate signaling (Lv *et al.*, 2014). This interaction is enhanced by the presence of nitrate and results in the degradation of OsSPX4 (Hu *et al.*, 2019). Furthermore, OsNLP3 (the functional ortholog of AtNLP7), the central transcription factor of nitrate signaling in rice, also possesses the ability to shuttle between the cytoplasm and nucleus to activate nitrate-responsive genes under nitrate stimulation. OsNLP3 physically interacts with OsSPX4 and this interaction blocks shuttling of OsNLP3 into the nucleus (Hu *et al.*, 2019). Thus, this work revealed a regulatory module, OsNRT1.1B–OsSPX4–OsNLP3, which fills the gap between nitrate sensing at the plasma membrane and the downstream nitrate response in the nucleus. Furthermore, NBIP1 (OsNRT1.1B Interacting Protein 1), a RING-type E3 ubiquitin ligase, was also identified, via immunoprecipitation followed by mass spectrometry, to underlie the mechanism of OsNRT1.1B-mediated OsSPX4 degradation (Fig. 2) (Hu *et al.*, 2019). OsNRT1.1B may function as a scaffold to recruit NBIP1 and promotes OsSPX4 ubiquitination and degradation when nitrate is available, resulting in the release of OsNLP3 into the nucleus to activate the downstream nitrate response. These findings therefore demonstrated that the OsNRT1.1B–OsSPX4 cascade mediates transduction of the nitrate signal from the plasma membrane to the nucleus to activate the downstream nitrate response (Hu and Chu, 2019).

## The OsNRT1.1B–OsSPX4 module integrates nitrate and phosphate signaling

Nitrogen and phosphorus are two important macronutrients for plants. They must be used in a coordinated manner for plants to achieve optimal growth and maximum yield in various natural environments as well as farmland systems (Gusewell, 2004; Lopez-Arredondo *et al*., 2013). Research on the OsNRT1.1B–OsSPX4 module also uncovered a mechanism for integrating the nitrate and phosphate signaling networks in rice (Hu *et al.*, 2019). Nitrate-triggered interaction of OsNRT1.1B with OsSPX4 promotes the degradation of OsSPX4 and the subsequent release of OsPHR2 (Zhou *et al.*, 2008), the central transcription factor of phosphate signaling, into the nucleus to activate the phosphate response. These findings thus established a link between nitrate stimulation and phosphate utilization, which contributes to our understanding of the molecular basis of the coordinated utilization of nitrogen and phosphorus (Fig. 2) (Hu *et al.*, 2019). The OsNRT1.1B–OsSPX4 module thus not only mediates the transduction of nitrate signaling from the plasma membrane to the nucleus, but also coordinates the nitrate and phosphate responses in rice, further demonstrating an important role of NRT1.1 in the integration of different nutrient signals (Hu and Chu, 2019; Poza-Carrion and Paz-Ares, 2019).

## OsNRT1.1B is involved in modulating the root microbiome in rice

As well as directly absorbing mineral nutrients via the roots, plants can interact with a wide range of microbes in the soil to facilitate the utilization of nutrients. Very recent work has demonstrated that OsNRT1.1B also plays an important role in determining the diversity of the root microbiome in rice (Zhang *et al.*, 2019*a*). OsNRT1.1B increases the populations of bacteria related to organic nitrogen mineralization in soil, which contribute to the transformation of organic nitrogen into nitrate or ammonium, in turn favoring nitrogen utilization (Fig. 2). Moreover, the natural variation in *OsNRT1.1B* is highly associated with differences in the root microbiome between *indica* and *japonica* rice, providing an explanation for the difference in NUE between the two subspecies. This work indicates that OsNRT1.1B can alter the rhizosphere microenvironment by modulating root microbes related to nitrogen transformation, and thus affect NUE. Further research should explore in depth the mechanism by which OsNRT1.1B regulates plant–microbe interaction, as this would provide new insight to understand the function of NRT1.1 in plants.

## Functional divergence of NRT1.1s in rice

There is a significant difference in the number of *NRT1.1* genes between monocots and eudicots, with grass species generally possessing three or four *NRT1.1* members while Arabidopsis has only one *NRT1.1* gene (Fig. 1) (Plett *et al.*, 2010). In rice, there are three homologues of AtNRT1.1, named OsNRT1.1A/OsNPF6.3, OsNRT1.1B, and OsNRT1.1C/OsNPF6.4 (Wang *et al.*, 2018b). OsNRT1.1B has a conserved function with AtNRT1.1: both show nitrate-inducible expression and plasma membrane localization, and are involved in nitrate uptake and transport as well as nitrate signaling (Hu *et al.*, 2015). Surprisingly, even though OsNRT1.1A shares the highest protein sequence similarity with AtNRT1.1 among the three rice homologues, it has a different function from the previously characterized NRT1.1s. In contrast to OsNRT1.1B, OsNRT1.1A exhibits ammonium-inducible expression and tonoplast localization, and can greatly up-regulate the expression of both nitrate and ammonium utilization-related genes, suggesting that it may play a vital role in modulating both nitrate and ammonium utilization in rice (Wang *et al.*, 2018*b*). Rice is traditionally viewed as an ammonium-preferring plant in the paddy field. However, rice root aerenchyma cells can release oxygen to the rhizosphere, where nitrification could occur, which could result in 25–40% of the total nitrogen taken up by rice being in the form of nitrate (Li *et al.*, 2008; Xu *et al.*, 2012). In addition, under a condition with mixed ammonium and nitrate supply, rice displayed better growth performance than with ammonium or nitrate supply alone (Kronzucker *et al.*, 1999). Thus, the functional divergence between OsNRT1.1A and OsNRT1.1B might be an important strategy for the environmental adaptation of rice. It might be speculated that the plasma membrane-localized OsNRT1.1B is responsible for sensing environmental nitrogen stimulation to trigger the downstream nitrate response, while the tonoplast-localized OsNRT1.1A may sense the intracellular nitrogen status to fine-tune the process of nitrogen utilization (Fig. 2).

## Future perspectives

With the functional characterization of NRT1.1 in different plants, our knowledge about NRT1.1 has increased dramatically, and it is gradually becoming apparent that NRT1.1 may not act simply as a nitrate transporter. The different paralogs in monocots have different nitrogen-responsive expression patterns and different subcellular localizations, which lead to their different mechanisms of regulating nitrogen utilization, but further work is needed to elucidate these mechanisms in greater details. For example, OsNRT1.1A may sense the intracellular nitrogen status (Fig. 2), but the underlying mechanism is unknown. OsNRT1.1A also regulates flowering time, but the mechanisms by which OsNRT1.1A is involved in regulating the expression of flowering genes such as *Hd3a*, *Ehd1*, and *RFT1* are also elusive. Future work concentrating on uncovering the regulatory mechanism of OsNRT1.1A will be helpful for answering these questions.

Notably, many transcriptomic studies have shown that responses to one nutrient are affected by the availability of other nutrients (Ueda and Yanagisawa, 2019). Considering the role of the OsNRT1.1B–OsSPX4 module in linking the downstream core transcription factors, OsPHR2 and OsNLP3, which respectively control the phosphate and nitrate response, it is possible that this module is also involved in the phosphate-mediated nitrate response, and OsNRT1.1B could function as an N–P integrator to sense external nitrate and phosphate. However, further efforts are needed to investigate the detailed mechanisms of this N–P integrator in sensing different levels of nitrate or phosphate signal, and the relevant downstream signal transduction pathway.

Both OsNRT1.1A and OsNRT1.1B have been shown to have great potential in crop improvement. *OsNRT1.1B* is highly diverged between the two subspecies of Asian cultivated rice, *indica* and *japonica*. The near-isogenic line with introgression of *OsNRT1.1B*^*indica*^ into *japonica* rice increases grain yield significantly (Hu *et al.*, 2015), suggesting that *OsNRT1.1B*^*indica*^ introgression has great potential to improve NUE in *japonica* varieties (Chao and Lin, 2015). Recent work has also revealed that overexpression of *OsNRT1.1B* significantly promoted selenomethionine transport from roots to shoots, resulting in the accumulation of Se in both shoots and grains (Zhang *et al.*, 2019*b*). This finding indicates that *OsNRT1.1B* has great potential in mineral biofortifcation, which provides a new way to produce selenium-enriched crops. Overexpression of *OsNRT1.1A* dramatically improves NUE and grain yield, and significantly shortens maturation time in rice (Wang *et al.*, 2018*b*). This provides a potential solution for cultivating high-yielding and early-maturing rice varieties to overcome the problem of ‘greedy green’ (in which the plant maintains a high chlorophyll content for an extended time even after it has matured) and late maturing caused by high nitrogen fertilizer in agricultural production. In recent years, base editing has emerged as a new genome-editing approach that could be used for improving crop varieties to meet future demands (Hua *et al.*, 2019; Mishra *et al.*, 2019; Zhang *et al.*, 2019*c*). A single nucleotide polymorphism in *OsNRT1.1B* between *indica* and *japonica* results in significant alteration of NUE; this suggests that precise single-base changes made via base editing could produce elite variants of agronomic traits in crops, which may facilitate NUE improvement. Deep exploration of genetic variation in *OsNRT1.1A* and *OsNRT1.1B* via base editing might be a powerful strategy for the further improvement of NUE and grain yield in rice.
